# Education of the Deaf and Dumb

**Published:** 1849-02

**Authors:** 


EDUCATION OF THE DEAF AND DUMB.

The Annual Report of the Kentucky Institution for the education of the Deaf and Dumb, for the year 1848, shows a prosperous state of things in this noble Charity. It is creditable to the Legislature of Kentucky that the institution is so well sustained, and to the benevolence and wise administration of its superintendents, that such results are exhibited in the annual reports.

